# Supplementary figures and images for: SPAG7 deletion causes intrauterine growth restriction, resulting in adulthood obesity and metabolic dysfunction
Source: eLife. 2024 Jul 26;12:RP91114. doi: 10.7554/eLife.91114 (PMC11281781; doi:10.7554/eLife.91114)

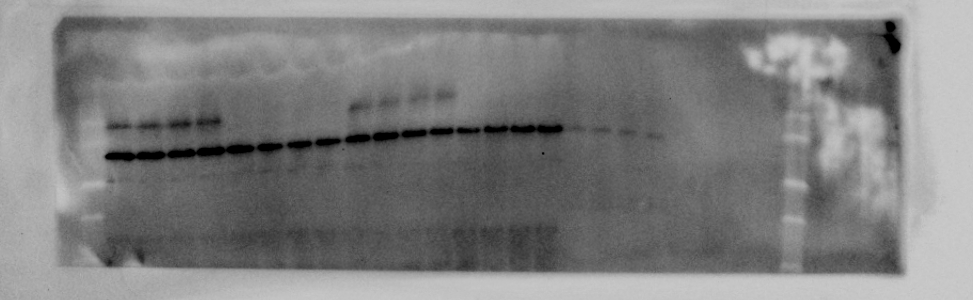

Supplement: Figure 4—source data 1. — Raw and annotated WB images. The representative western blot images for 4B are indicated within a blue square. [file elife-91114-fig4-data1.zip › Figure 4-source data 1/Fig 4B Liver and Brain SPAG7 and bACTIN copy.png]

Figure 4B

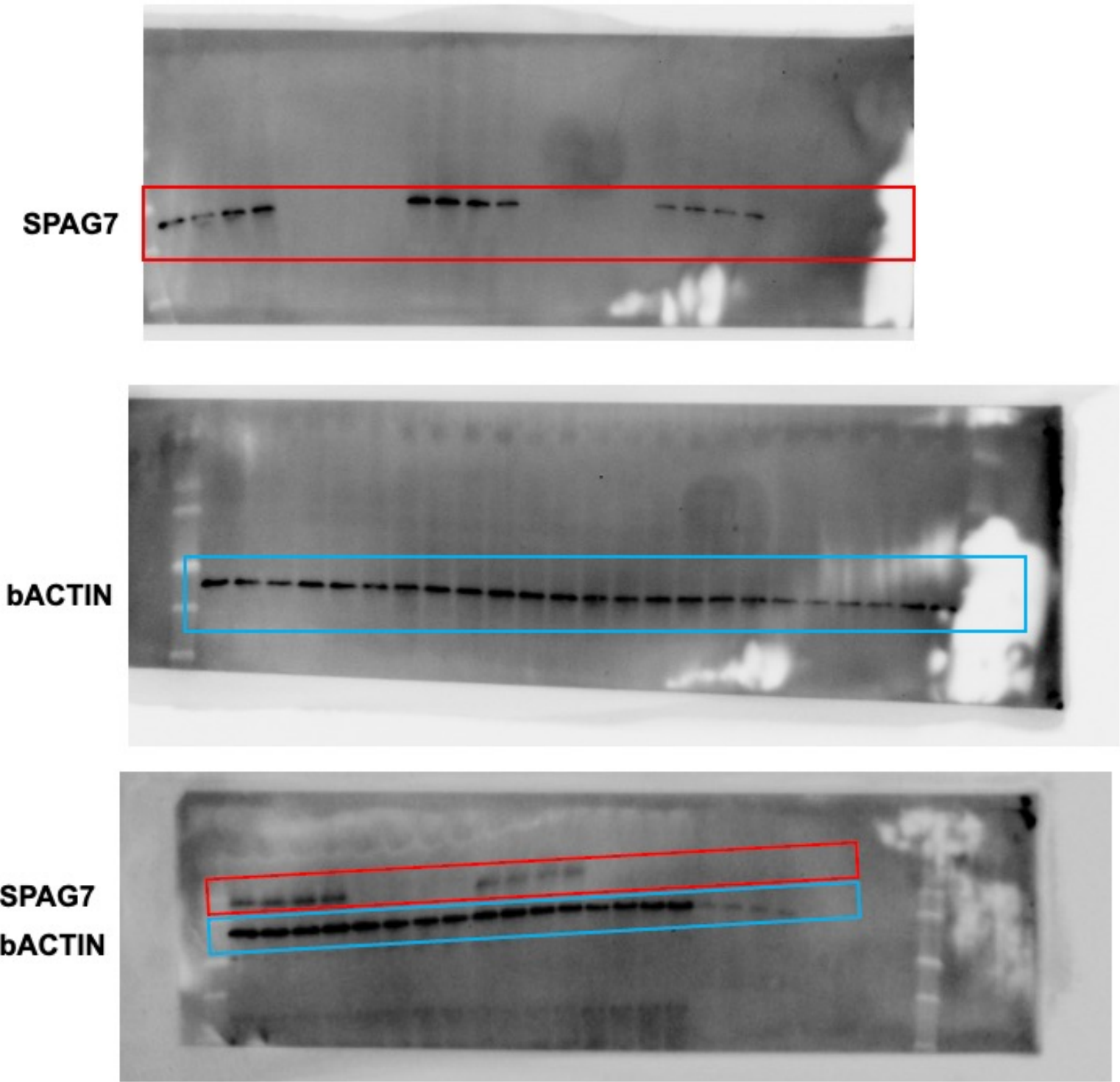

Supplement: Figure 4—source data 1. — Raw and annotated WB images. The representative western blot images for 4B are indicated within a blue square. [file elife-91114-fig4-data1.zip › Figure 4-source data 1/Source Data_Figure 4.pdf]

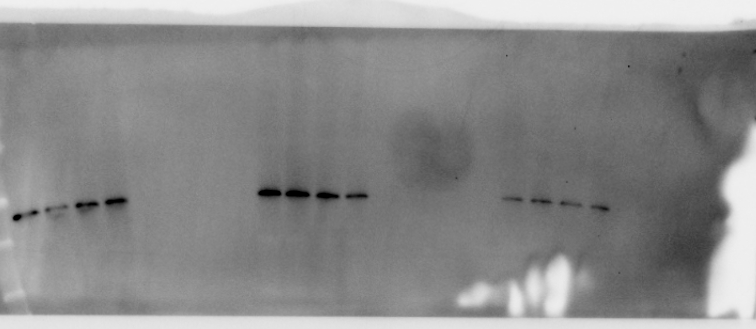

Supplement: Figure 4—source data 1. — Raw and annotated WB images. The representative western blot images for 4B are indicated within a blue square. [file elife-91114-fig4-data1.zip › Figure 4-source data 1/Fig 4B Kidney Gastroc PGAT SPAG7.png]

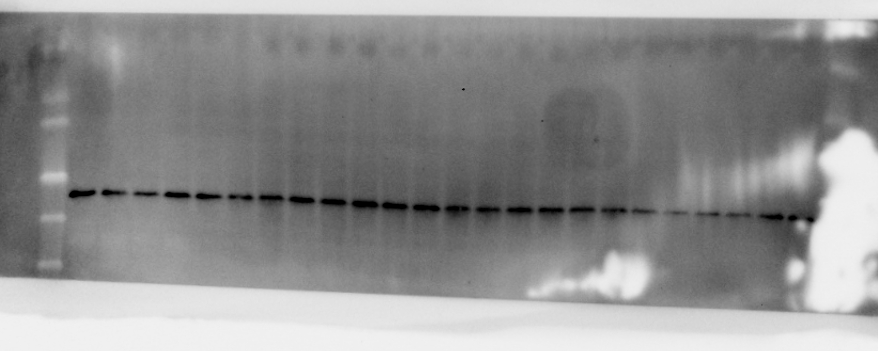

Supplement: Figure 4—source data 1. — Raw and annotated WB images. The representative western blot images for 4B are indicated within a blue square. [file elife-91114-fig4-data1.zip › Figure 4-source data 1/Fig 4B Kidney Gastroc PGAT bACTIN.png]

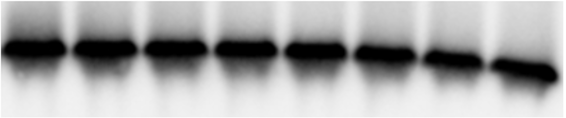

Supplement: Figure 5—source data 1. — Raw and annotated WB images. The representative western blot images for 5 M are indicated within a blue square. [file elife-91114-fig5-data1.zip › Figure 5-source data 1/Fig 5M bACTIN.png]

Figure 5M

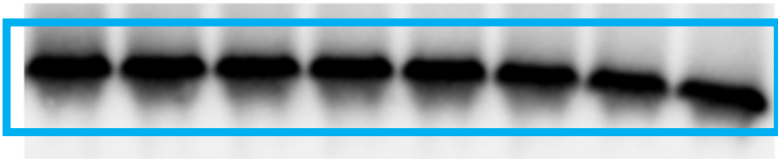

bACTIN

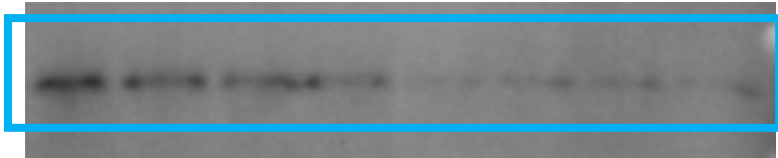

IGF1

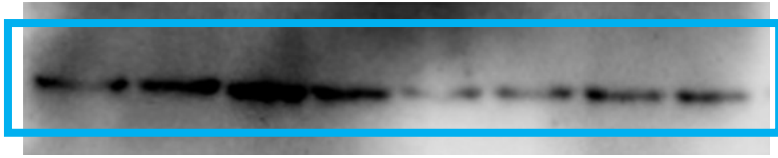

IGF2

Supplement: Figure 5—source data 1. — Raw and annotated WB images. The representative western blot images for 5 M are indicated within a blue square. [file elife-91114-fig5-data1.zip › Figure 5-source data 1/Source data_Figure 5.pdf]

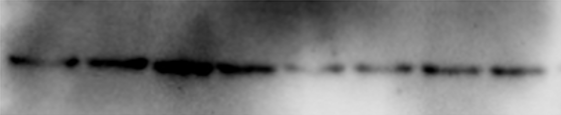

Supplement: Figure 5—source data 1. — Raw and annotated WB images. The representative western blot images for 5 M are indicated within a blue square. [file elife-91114-fig5-data1.zip › Figure 5-source data 1/Fig 5M IGF2.png]

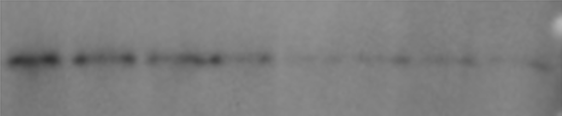

Supplement: Figure 5—source data 1. — Raw and annotated WB images. The representative western blot images for 5 M are indicated within a blue square. [file elife-91114-fig5-data1.zip › Figure 5-source data 1/Fig 5M IGF1.png]
